# Exploring the Potential Causal Relationship Between Health Insurance Coverage and Child Nutritional Status in Pakistan: Evidence from PDHS-2018

**DOI:** 10.3390/healthcare13050532

**Published:** 2025-02-28

**Authors:** Muhammad Shahid, Zaiba Ali, Subuhi Khan, Muhammad Shahzad Yousaf, Zhe Zhang, Jiayi Song

**Affiliations:** 1 School of Insurance and Economics, University of International Business and Economics, Beijing 100029, China; de202159006@uibe.edu.cn; 2 Department of Management, College of Business Administration, Princess Nourah Bint Abdulrahman University, P.O. Box 84428, Riyadh 11671, Saudi Arabia; zaiali@pnu.edu.sa (Z.A.); szkhan@pnu.edu.sa (S.K.); 3 School of International Development and Cooperation, University of International Business and Economics, Beijing 100029, China; shahzad.mehr@numl.edu.pk; 4 School of Economics, Renmin University of China, Beijing 100872, China; 5 School of Healthcare Management, Tsinghua University, Beijing 100190, China

**Keywords:** child health, health insurance coverage, nutritional status, binary logistic regression, propensity score matching, PDHS

## Abstract

**Objectives:** the current study investigates the link between health insurance coverage and child nutritional status in Pakistan. **Methods:** Using data from the Pakistan Demographic and Health Survey (PDHS) 2017–18, encompassing 4499 children under 5, a binary logistic regression was applied to analyze the relationship between health insurance and child nutritional status. Due to the non-randomized sample, assessing health insurance continuously posed a practical challenge. To mitigate the sample selection bias, the cross-sectional-based propensity score matching (PSM) using the nearest neighbor method was utilized for the causal relationship, based on potential socio-economic covariates. **Results:** The prevalence rates of stunting, underweight, and wasting among children under five were 38.13%, 23.04%, and 8.05%, respectively. Malnutrition was found in 43.64% of non-insured children compared with 5% in insured children. The findings of PSM supported a causal relationship, given the cross-sectional nature and potential misplaced variables, as the PSM findings revealed that insured children had significantly better nutritional outcomes compared with non-insured children, with a significance level of 1%. The logistic regression outcomes for the covariates of child nutritional outcome indicated that health insurance coverage, higher wealth status, mother’s education, improved water and sanitation facilities, mother’s normal BMI, and urban residence reduced the likelihood of child malnutrition. The logistic regression results for the covariates of child health insurance depicted that factors such as higher birth order, mother’s low BMI, poor water and sanitation facilities, higher wealth status, women’s employment, higher education level, and child illnesses like diarrhea and malnutrition increased the likelihood of obtaining health insurance. The logistic results confirmed that health insurance coverage reduced the likelihood of child malnutrition, and, similarly, child malnutrition and other illnesses increased the chances of obtaining health insurance coverage. **Conclusions:** The findings underscore the critical need for health insurance, highlighting its role in enhancing child nutritional status. The government should expand health insurance programs, with a special emphasis on child nutrition and health.

## 1. Introduction

Healthcare is considered a fundamental right and an essential need for the residents of any nation [1]. The development of a country and the general well-being of its population significantly depend on the availability of essential healthcare services. In developing nations, access to these services remains severely limited. The availability and accessibility of critical healthcare facilities are major issues in these regions, where poverty and inequality significantly impact individual health, care accessibility, and health-related services. Recently, developing countries have critically implemented non-profit health insurance programs that provide access to essential healthcare services without imposing financial stress on the population [2,3].

In recent years, health insurance coverage has emerged as a crucial tool in low- and middle-income countries to enhance equity in the delivery of medical care services [4]. Similarly, one of the national insurance programs in Pakistan is the Ehsas Sehat Sahulat program. The primary goal of this insurance program is to ensure that every citizen has access to affordable and high-quality medical care services [5,6,7]. It reduces economic barriers and provides financial support to marginalized households for medical treatment and healthcare services [2]. Every year, approximately 100 million individuals fall into poverty due to the distress of medical care expenditures, meaning that 50% of the global population still lacks access to essential healthcare services [8]. Pakistan’s healthcare system currently ranks 154th out of 195 nations. As a developing country, Pakistan allocates only 2% of its GDP to health-related expenses, struggling to maintain an adequate healthcare system in terms of standards, affordability, and quality [9]. Despite having the lowest health insurance coverage in the region, Pakistan’s National Health Vision 2016–2025 aims to address the needs of those suffering from diseases. The primary reasons for the lack of awareness regarding healthcare insurance are the high rates of poverty and illiteracy. Consequently, Pakistan falls below the ideal level of health-related insurance coverage.

According to the DHS assessment, 1.77% of women and 2.36% of men report having health insurance in Pakistan. Additionally, 1.67% of children under five years old are reported as having health insurance. In Punjab, the MICS assessment shows that 3.2% of women and 3.9% of men have health insurance, while only 2.3% of children under five have access to healthcare insurance. Despite these low figures, Pakistan has made significant efforts to improve health services. However, these efforts are impeded by various management challenges and resource shortages, which affect the delivery of healthcare services in the community [10]. People are hesitant to purchase health insurance services due to their awareness of the healthcare conditions and rising costs. A significant portion of Pakistan’s population has not recognized the necessity of obtaining health insurance, primarily due to poverty and lack of awareness [11]. Furthermore, many people believe that young individuals are unlikely to be affected by acute diseases and that, if illnesses do occur, they can manage medical expenses on their own rather than paying premiums for years. However, health insurance is crucial for everyone, regardless of age, because illness can strike anyone at any time, and, without insurance, rising healthcare costs can burden the entire household.

For older individuals, investing in nutrition and healthcare services for children is particularly important and effective. Children with better health and nutrition contribute to the overall development of the community. Additionally, good childhood health significantly impacts educational and labor outcomes later in life [12,13,14,15]. It has been universally found that one in every three poor children faces malnutrition due to a lack of access to education, water, sanitation, and healthcare [16]. To some degree, improving the health standards of children can be achieved through health insurance, which is seen as a feasible option. Evidence shows that the well-being of a child and health insurance are positively correlated [17,18,19,20]. The provision of basic health insurance, along with other support services such as mandatory charity for poor Muslims known as zakat (donation) and general charity, can effectively address issues related to poor health, nutritional outcomes, and poverty [21,22].

Moreover, underdeveloped countries like Pakistan are grappling with malnutrition, a severe health problem affecting at least half of its children under the age of three [23,24,25]. Among these concerns, underweight, stunting, and wasting are common in children [26]. The highest prevalence of these conditions is found in children from Asian and African regions [27]. All types of undernourishment in countries such as Pakistan, India, and Bangladesh exceed the satisfactory thresholds of 15% wasting, 10% underweight, and 30% stunting [27,28,29]. Children with malnutrition concerns have a 4–12 times greater risk of mortality compared with healthy children [30].

Previous studies have identified this phenomenon, but there is a notable lack in the literature specifically addressing the causal relationship between health insurance and child nutritional status in Pakistan. In-depth empirical studies examining the association between health insurance coverage and child nutritional status at a national level in Pakistan are surprisingly sparse. This study aims to fill this gap by considering the entire country as a case study using data from the Pakistan Demographic and Health Survey (PDHS). The present research is intended to serve as a baseline scenario for health insurance coverage, assisting policymakers and planners in designing strategies for expanding health insurance in Pakistan to improve the nutritional status of children. Recognizing that the future of any nation depends on its children, this study underscores the importance of health insurance and child nutrition. By utilizing national data from the PDHS, this study investigates the potential causal relationship between child nutritional status and health insurance. The findings will offer insights for improving child nutrition and effectively using health insurance to achieve universal health coverage. This research will contribute to the literature on children’s nutritional status and health insurance coverage in Pakistan.

## 2. Method and Materials

### 2.1. Theoretical Framework of This Study

Many studies conducted on child malnourishment assessment follow the utility maximization model by hypothesizing the household-making function [31,32]. The utility maximization model undertakes that each child lives in a unit known as a house and every family increases their utility by performing the following:

(1) U=U [L, X, Ni]

The above equation specifies that a household is composed of three main elements: the consumption of a variety of items [X], the allocation of leisure time [L], and adequate nutrition quality [Ni] for children. Ni is used as a symbol for measuring the underweight (WAZ), wasting (WHZ), and stunting (HAZ) anthropometrics of children. Moreover, the nutrition status of a child, as denoted by [Ni], is influenced by various social and economic variables, such as:

(2) Ni=n [HSF, CON, H, CM, ε]

For a child, Ni is used using a regular assessment of the child’s physical anthropometry. Where CON depicts intake, the vector of particular children plus motherly elements is represented by CM; the vector of particular household socio-economic factors is represented by HSF; the vector of health factors is represented by H, and the symbol ε represents the error term of a particular child. This equation depicts the entire impact of a child’s undernutrition in terms of its causing factors.

For this study, the decreased pacificated type of nutrition production function is:

CIAFi=f(house socio−economic elements, illness or health−related factors, specific factors related to child and maternal, ε CIAF)

Moreover, this study also assesses the correlations of children having health insurance coverage. So, following the concept note of the above CIAF equation, this study uses this concept for correlations of health insurance. Below is the reduced form of children having health insurance coverage:

Health Insurance=f(socioeconomic factors, disease/health factors, child and maternal factors, ε Health Insurance)

### 2.2. Conceptual Framework

**Figure 1:**
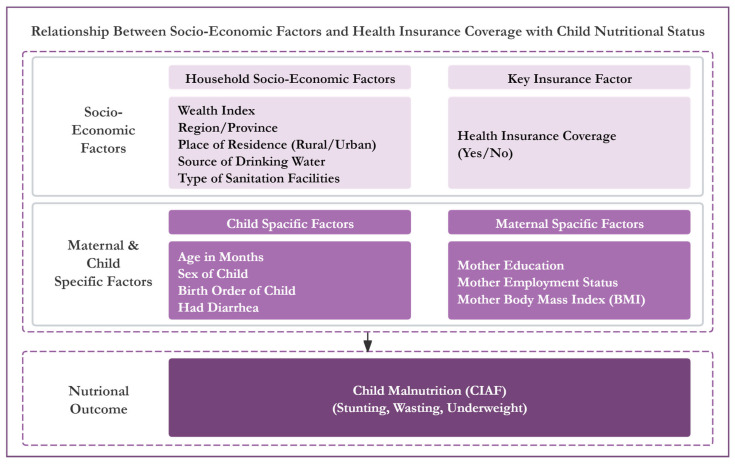
The current study’s operational conceptual framework. Source: authors.

Figure 1 depicts the operational conceptual structure of this paper.

### 2.3. Main Explanatory Variables

The contributing factors of child malnutrition and the correlation of children having health insurance coverage are assessed in this study. In the first model for the contributing factors of child-related malnutrition (CIAF), health insurance coverage (yes/no) is the main key explanatory variable. To define health insurance, a dummy variable is created by assigning dummy coding [1 = if any child is recovered by health insurance, while 0 = child has no insurance].

Health insurance is likely to be linked separately to other particular contributing factors. Hence, it is recommended to utilize the covariates to match the treatment set by the similar unit’s equivalent to the control group [33]. This case comprises the process of linking non-insured to insured children who are similar to one another. However, they differ merely in the level of insurance. In the present study, researchers established an initial estimate based on matching the demographic characteristics of each household with child insurance and without child insurance. And, in the first logistic model, some of the socio-economic factors, health insurance, and health conditions are taken as child nutritional status covariates. In the second logistic model, child nutritional status (CIAF) is used as an explanatory variable along with a covariate for health insurance.

### 2.4. Dependent Variables

This study uses two models. In the first model, the dependent variable is CIAF (cumulative index of anthropometric failure). This study focuses on children’s height and weight in addition to age to build indices reflecting child malnutrition from 2017–18 PDHS data. Following the World Health Organization standards for child growth (2009), three indices are employed [34]: (i) stunting (HAZ), well-defined by way of z-scores of height-for-age < −2 S.D. of the median value according to WHO rules; (ii) WAZ, defined as z-scores of weight-for-age < −2 S.D. of the median value according to WHO criteria; and (iii) wasting, defined as z-scores of weight-for-height < −2 S.D. of the median value based on WHO guidelines. This study developed the composite index of anthropometric failure (CIAF) classification, categorizing children into seven groups: (1) no child failure case, (2) merely stunting child case, (3) merely wasting child case, (4) merely underweight, (5) both child cases (stunting plus underweight), (6) both child cases wasting plus underweight), and (7) child having three conditions at the same time (stunted, wasted, plus underweight). In conclusion, the overall prevalence of child undernutrition is estimated by considering the sum of all categories leaving group A case. The A binary dependent variable is assigned the value “1” if a child is malnourished and “0” if a child is not malnourished.

In PDHS, the primary question is asked whether a household has health insurance coverage or not. In the second model, the main dependent variable considers the child health insurance coverage. Binary coding is performed for describing the child health insurance coverage [1 = if a child receives treatment by health insurance, and 0 = if the child is non-insured].

In Pakistan, the federal government took an initiative in 2015 by launching the main Sehat Sahulat national health insurance scheme for the nation. The purpose of this national insurance program was to give 100% free indoor health services using Benazir Income Support Program (BISP) data from the poor segment of society, as it was meant for the poorest of the poor. All types of general medicine (especially diabetes, hypertension, cardiac), in patient services (all medical and surgical procedures), emergency treatment requiring admission, maternity services (normal delivery and C–section), burns, and road accidents are covered by the Sehat Sahulat national health insurance program. When a family is registered in the national health insurance program, their children are automatically considered as registered based on their own and the head of household national identification cards (IDs). So, the Sehat Sahulat national health insurance scheme has recently covered the entire poor population in major provinces in Pakistan. So, in the PDHS data, of the households that are insured, most of them are registered in the Sehat Sahulat national health insurance scheme. So, for that reason, we only took one variable for this study, which, in binary form, is “household is covered by health insurance (Yes/No)”.

### 2.5. Description of the Dataset

The current investigation utilizes records from the PDHS carried out in 2017–2018, encompassing 4499 children under the age of 5. The dataset provides comprehensive knowledge on nutrition, demographics, women’s empowerment, domestic violence, and various healthcare aspects related to women and children. Anthropometric measurements of eligible children under three years of age are employed for statistical analysis. Additionally, household socio-economic characteristics, health insurance coverage, child- and mother-specific elements, and factors of child disease are considered for the determinants of child malnutrition and health insurance coverage.

### 2.6. Public and Patient Involvement

This study’s participants or respondents are not included in the design, conduct, writing, or distribution strategies of this investigation.

### 2.7. Statistical Analysis

This study employs logistic regression to investigate the link between the socio-economic coverage of health insurance variables and the predicted variable, which is child undernutrition (CIAF). Logistic regression calculates the probability or probability proportions of the resulting variable (CIAF) based on descriptive factors. The simplified form of the logistic model is provided as:

(3) P(Yi=1|X1i,X2i,…,Xkn)=F(β0+β1X1i+β2X2i+…+βnXkn)

The outcome variable (CIAF) is denoted by Yi in the equation above, while Xi stands for the descriptive variables, and the coefficients stand for “βs”, which describe the strength of the connection to CIAF, and the error form is “ε”.

Furthermore, as found in the studies on health insurance, in the present study, researchers also face a main methodological problem of sample selection bias. Thus, to eliminate the worry that alike aspects can drive health outcomes and health-related insurance, the method of propensity score matching is carried out in this study. As stated by Nichols, confounding variables are measured directly for assessing causal inference of effects by employing conventional regression [17]. But it is also addressed that, when the covariates link with the endogenous (residual), selection biases can occur [17,18]. If, because of only observed characteristics, selection bias of the sample appears and all observed confounders are included in the model, it probably affects the child’s health and the health insurance of the child. In this kind of situation, the most appropriate tool to use is the propensity score method. The propensity score method largely applies to reaching the influence of treatments when based on non-experimental data [35]. For each untreated and treated participant, the propensity score is considered in addition to matching participants from the treated set with one or several participants from the untreated set having similar propensity scores. Several noticeable dissimilarities are decreased to a dissimilarity of one element with the PSM approach [36]. The propensity score method’s foremost objective is to reach the total treatment impact regarding child health insurance coverage, established based on the average treatment influence on the treated (ATT). Thus, the present study implements the following key steps to empirically explore the phenomenon, and the logistic methodology is used to calculate the propensity score value (fitted value) of the conditional possibility of the children with health insurance:

(4) PSM=Pr[LM=1|XM]=E[LM=0|XM]

where LM = 1 denotes health-insured children (considered as the treated group), and LM = 0 denotes non-insured health children (considered as the control group); XM indicates the observable covariates (like economic conditions of the family, mother, and head of the house). Based on possible covariates, both the control and the treatment groups are matched. This study’s authors employ the k nearest neighbor matching method, which is established by matching sample participants by finding k persons from dissimilar sets with the nearest propensity score to increase the reliability. To reduce the error of mean square, one-to-one matching is implemented and k is set to 1 in the present study.

To sum up the health level dissimilarity between the control and treatment groups, the ATT method is applied. After this, the impact of a child’s health insurance on the well-being of the child is finally obtained.

(5) ATT=E[D1M|LM=1]−E[D0M|LM=1]=E[D1M−D0M|LM=1]

D1M represents insured children’s nutritional status, D0M represents non-insured children’s health status, E [D1M|LM = 1] might be assessed straightly, and E [D0M|LM = 1] cannot be assessed straightly; in addition, it will be a counterfactual finding. Accordingly, PSM is an appropriate method for generating the matching auxiliary index.

## 3. Results

To gauge the sensitivity or responsiveness to change in CIAF due to predictors of malnutrition, Table 1 provides the percentage estimation of malnutrition. The table reveals that malnutrition occurrence is slightly higher in male children (22.49%). The prevalence rates are elevated in the 25–36- and 37–48-month age groups (10.43%). Malnutrition is more prevalent in birth orders less than or equal to 2 years (18.71%). Children who recently had diarrhea exhibit a higher malnutrition prevalence (35.04%). Rural areas in Pakistan bear a higher burden of malnutrition (26.23%). The malnutrition rates are elevated in children from Punjab, Sindh, KPK, and Balochistan, at 7.25%, 10.16%, 7.03%, and 7.45%, respectively. Malnutrition prevalence is higher (38.58%) among children whose mothers are not employed. Illiteracy in mothers is associated with higher malnutrition rates (27.99%). Malnutrition rates are elevated (39.62%) among children whose mothers are underweight or have a low body mass index. Children from the poorest and poorer wealth status households have higher prevalence rates, at 13.43% and 12.38%, respectively. Malnutrition prevalence rates are high (39.62%) among children with an unimproved water source in their homes. Rates are higher (31.36%) among children with unimproved sanitation facilities. Malnutrition rates are highest (43.64%) among children from households without any health insurance.

### 3.1. The Impact of Covariates on Nutritional Status of Child

Table 2 displays the results of binary logistic regression for the determinants of child malnutrition. Notably, an increase in the age of children corresponds to greater chances of undernutrition in children under five. Similarly, as birth order increases, the likelihood of child malnutrition also rises. Urban households exhibit a lower prevalence of malnutrition compared with rural ones. Regions such as Sindh, KPK, Balochistan, and FATA show higher chances of undernutrition in children under five. Children are less likely to be malnourished when their mothers have a normal BMI. Chances of malnutrition are greater in children below the age of five who have recently had diarrhea. Mothers with secondary and higher education have lower probabilities of malnutrition in their children compared with those with primary and no education. Children under five whose mothers are working have higher odds of malnutrition compared with those whose mothers are not employed. Households with better quality water sources and improved hygiene amenities have lower odds of malnutrition in their children compared with those with unimproved facilities. Crosswise wealth quantiles and probabilities of undernutrition are less in middle, richer, and richest households. Children with health insurance coverage exhibit lower odds of malnutrition compared with those without insurance. It shows that malnutrition prevalences are lower in those children who have health insurance coverage.

### 3.2. Influence of Covariates on Child Health Insurance Coverage Through Logistic Regression

Health-insured children are likely to obtain healthcare in time, and they are less likely to be exposed to malnutrition and to be hospitalized compared with non-insured children. However, numerous factors such as socio-economic factors might hinder the approach to health insurance. Consequently, children’s health insurance coverage is mainly evaluated by using a multivariate logistic regression model established on socio-economic and child health confounders. The results are discussed in Table 3.

The logistic results reveal that increasing the number of children or birth order increases the probability of acquiring health insurance. Households belonging to rural places lower the chances of receiving health-related insurance coverage. Chances of receiving any health insurance coverage are greater in KPK, Islamabad, and Gilgit Baltistan compared with lower probabilities of receiving health insurance in Sindh and Balochistan provinces. Children having illnesses like diarrhea or malnutrition have higher probabilities of attaining health insurance. Unimproved drinking water plus sanitation facilities increase the odds of receiving health insurance coverage. Families belonging to richer and richest families have higher odds of receiving health insurance. A mother’s low BMI increases the chances of acquiring health insurance. The odds of health insurance increase if women have employment status. The higher education of mothers increases the chances of attaining health insurance coverage.

### 3.3. Common Support Domain

**Figure 2:**
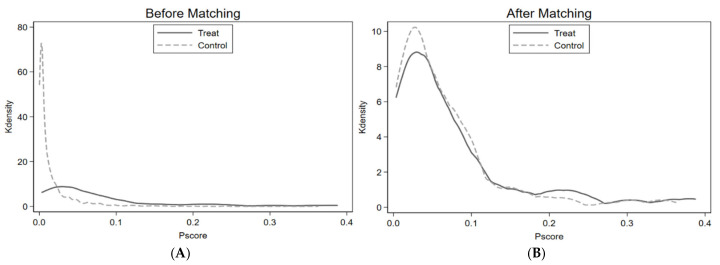
Propensity score distribution before and after matching. (**A**) Before matching. (**B**) After matching.

The overlay in values between the treatment group and the control group is revealed by the common support areas that are identified in the first step of PSM. To confirm matching qualities, Figure 2 shows the zone of common support for the control and treatment groups. The graphs in Figure 2 also represent the function density of PSM before and after. This shows the overlap between the PS values of both insured and non-insured children. Therefore, the authors of this study believe that the maximum number of data information or observations fall within the common support range values. So, within this investigation, employed data have improved the situations for the common support domain. Furthermore, as per the outcomes of this study, the loss of samples is minor, as presented in Table 4; concerning these, 198 and 3 samples are lost in both the control and treatment groups, leaving a total of 3540 matched samples.

### 3.4. Balance Test: Nearest Neighborhood Matching

The balance test is applied in the next step. The objective of performing this test is to reach the standard of matching through likening consequential covariates between the treatment and control groups. Those participants whose propensity score is not within range of the tendency scores of the control group (people belonging to the not common support area) are eliminated from the test. Table 5 presents the balancing properties’ total matching quality signs. After matching, the standard deviation decreases from 21.4 to 10.7 for the two groups (treated and control). The findings reveal that the quality of matching is adequate because the overall bias significantly decreases. The test of joint significance covariates, at 1%, is highly significant. Moreover, this analysis shows that the value of pseudo-*R*2 decreases from 0.190 (before matching) to 0.015 (after matching). Thus, there is no substantial dissimilarity between health-insured and non-insured children. The distribution of covariates between the treatment and control groups is effectively balanced by the matching method and the selection bias of the sample is successfully reduced in the present investigation.

### 3.5. Before and After Matching Covariate Balance Findings Between Treated and Untreated Groups

Table 6 shows the unmatched and matched groups’ summary statistics for all covariates included in this study. In columns (3 to 6), standardized bias percentage (%), bias reduction percentage (%), and *t*-value statistics have significant levels, while, in the first two columns, the means of the group are presented for the comparison of treated groups. For the incomparable sample, the control and treatment groups’ covariate mean for each covariate are compared in the first row. The table representing the covariates between the control and treated groups differ significantly and the significance of all covariates are problematic at the time of measuring the treatment effect. The mean statistics balance and differences show no systematic differences in the control and treated groups after matching and also most of the covariate’s groups are balanced (for each covariate second row) [24,37]. For most covariates, the standardized difference is extremely substantial at a 1% level of significance, and balance findings commonly propose the suitability of measuring the ATT by performing a comparison of two sets.

### 3.6. Treatment Effect Estimation/Average Treatment Effect (ATT)

Eventually, the treatment influence of health insurance (predicted variable) on the child’s health/nutritional level (outcome variable) is examined and stated in Table 7. By using the method of the nearest neighbor, ATT measures a report that reports a substantial influence at a significance level of 1%. This is a positive digit, after improving the choice bias of sample results shows that health insurance significantly enhances children’s health.

## 4. Discussion

The primary objective of this paper was to determine the causal effect of health insurance coverage on the nutritional status of children, using data from the PDHS 2017–18. Propensity score matching (PSM) was utilized to assess the causal effect of health insurance on child nutritional status. It is considered that children with health insurance are more likely to receive early medical care compared with those without insurance. However, access to health insurance can be hindered by numerous barriers. Therefore, propensity score matching was employed in this investigation. By applying this method and estimating the average treatment effect, this study utilized logistic regression to evaluate the impact of different socio-economic factors on child nutritional outcome, as illustrated in Table 2. This research then used another logistic model to approximate the impact of various socio-economic covariates that may influence insurance coverage, as shown in Table 3. The overall prevalence of malnutrition was approximately 44.15%, which included stunting at 38.13%, underweight at 23.04%, and wasting at 8.05%. Additionally, the prevalence of malnutrition among children from non-insured families was around 43.64%.

The conversation surrounding socio-economic factors affecting child nutrition and health insurance coverage is crucial. Findings indicate that, as children’s age increases, so does the likelihood of malnutrition. However, age does not impact their health insurance coverage. These results align with previous studies [38,39]. Additionally, the data show that the likelihood of a child being undernourished is associated with higher birth orders. The research has indicated that having multiple siblings or being born later in the birth order can be a risk factor for child malnutrition [40,41]. Additionally, the results showed that an increasing number of children or higher birth order is associated with a greater likelihood of obtaining health insurance. The findings also indicated that children experiencing diarrhea are less likely to be undernourished. This study’s results align with other research, which confirms that children suffering from diarrhea have a higher occurrence rate of malnutrition [42,43]. Furthermore, children with illnesses such as diarrhea or malnutrition are more likely to obtain health insurance. Children who have diarrhea might need urgent healthcare, which leads to the need to acquire health insurance. Those with health insurance are more likely to access healthcare facilities, leading to a reduced susceptibility to illnesses.

Children from the Sindh, Balochistan, KPK, and FATA regions face a higher risk of malnutrition. Additionally, the likelihood of obtaining health insurance is greater in KPK, Islamabad, and Gilgit Baltistan, while the odds are lower in Sindh and Balochistan. There is a significant regional variation in nutrition across Pakistan. One study indicated that children in Sindh and Balochistan experience higher levels of malnutrition during their middle years compared with those in Punjab and KPK [44]. Another study also found that malnutrition prevalence is greater in Balochistan and Sindh compared with other provinces of Pakistan. This disparity is attributed to the lower socio-economic development in these regions [45]. The findings of this research indicate that the likelihood of malnutrition is lower among children born in urban areas compared with those in rural regions. Additionally, children in rural areas have a reduced chance of obtaining health insurance coverage. The results show that the prevalence of malnutrition is higher in rural areas than in urban areas, with urban children exhibiting a lower frequency of stunting [46]. This may be credited to better access to timely healthcare services and appropriate nutrition in urban settings. Consequently, children in rural areas of developing countries are considered more vulnerable to malnutrition due to limited economic resources and access to health services [47,48]. There are notable differences in infrastructure and development between rural and urban regions, such as the availability of roads and health services, which can affect access to medical insurance and healthcare. Many individuals eligible for health insurance programs live in rural areas and often must travel long distances to reach district card distribution offices. This distance, along with transportation costs, poses great barriers to accessing health insurance. Therefore, children with health insurance do not have to pay an annual premium; they only need to register at their local district offices. The results indicate that both secondary and higher education levels of mothers reduce the likelihood of child undernutrition. Additionally, mothers with higher education are more likely to obtain health insurance for their children. One study highlighted that maternal education, particularly related to health knowledge, plays a crucial role in child health [49]. This underscores the significant policy implications of focusing on maternal education in relation to children’s nutritional status.

While policymakers often prioritize primary education and adult literacy programs, it is fundamental to emphasize the importance of secondary education for mothers regarding children’s nutritional outcomes. Therefore, improving access to secondary education for all citizens should be a priority in Pakistan. Furthermore, mothers with higher education levels tend to contribute more to household income and are better equipped with knowledge about child feeding practices and health, which helps lower the prevalence of child malnutrition. Previous research supports these findings, concluding that a mother’s educational status is a significant factor influencing malnutrition [50,51]. Higher education also keeps women informed about societal issues and facilitates access to medical insurance for their children.

The results indicate that a woman’s employment status significantly lowers the odds of malnutrition in her children. Additionally, the level of employment for women increases the likelihood of acquiring health insurance for their children. One study suggested that a mother’s employment may impact childcare and nutrition, as working mothers may spend less time directly caring for their children [52]. However, the employment status of mothers also plays a crucial role in improving child nutrition. Women’s income enhances the family’s ability to afford healthy food and meet essential living needs. This study found that the employment status of mothers significantly reduces the risk of child malnutrition. These findings align with previous research [53]. Employment empowers women to make financial decisions for their families and increases their awareness of the benefits and importance of health insurance for better health outcomes. Employed mothers are more likely to make informed choices regarding health insurance coverage for their children.

This study’s results indicate that low BMI in mothers increases the risk of child malnutrition. Interestingly, the findings also show that mothers with low BMI are more likely to acquire health insurance coverage for their children. Healthy mothers are crucial for delivering healthy offspring; however, women with low BMI who are malnourished are at risk of giving birth to low-weight children, particularly in severe cases. Such children are more likely to experience starvation during infancy [54]. Specifically, mothers with a BMI of less than 18.5 kg/m^2^ have a higher likelihood of having low-birth-weight children [54]. The findings reveal a significant correlation between maternal BMI and malnutrition in children under five, aligning with previous research that shows a low maternal BMI increases the risk of child malnutrition [53]. Poor maternal health can render mothers more susceptible, adversely affecting their children’s health and development. Low maternal BMI may be a factor for maternal awareness of future health liabilities for them and their children. Consequently, addressing the health challenges faced by both mothers and children can promote a greater uptake of health insurance coverage.

Additionally, the results indicate that improved drinking water and sanitation services lower the risk of malnutrition among children. Conversely, inadequate drinking water and sanitation services are linked to a higher likelihood of obtaining health insurance coverage. Studies conducted in Pakistan have emphasized that access to safe water and sanitation is a crucial predictor of malnutrition [42,43].

Furthermore, this study illustrates that a greater wealth status reduces the likelihood of malnutrition in children. Previous research in Pakistan has shown that a lower economic status is a significant contributor to child malnutrition [55,56,57]. The findings of this paper highlight the significant impact of health insurance coverage on children’s nutritional outcomes. After adjusting for observable covariates, the influence of health insurance on children’s nutritional levels is found to be meaningful, indicating a substantial effect in Pakistan. Health insurance helps alleviate financial constraints related to medical expenses, making healthcare more accessible for families [58].

At the community level, various health and insurance programs have proven effective in reaching vulnerable populations by addressing financial, geographical, and socio-cultural barriers [59]. Such initiatives can enhance the ability of low-income individuals to afford medical treatment. Additionally, research has shown that conditional cash transfer programs can help reduce stunting in children under five children [60]. A study conducted in New York, USA, found that children’s health insurance programs significantly improved the health standards of children [61]. Studies from Ghana (DHS-2008), Rwanda (DHS-2010), and Indonesia (2012), taking cross-sectional data, using PSM, demonstrate that increased national health insurance coverage positively impacts the health of mothers and children [62,63,64]. In Indonesia, cross-sectional research conducted during the period from January to September 2019, using logistic regression, indicated that the Jaminan Kesehatan Nasional program significantly helps prevent undernutrition [65]. Similarly, a cross-sectional study conducted in rural Uganda from August 2015 to April 2016, using an instrumental variable approach, found that child stunting decreases by 4.3% in households participating in community health insurance programs for over a year [66]. Numeric data from the USA show that health insurance improves children’s health and reduces long-term reliance on community safety nets [67].

In Pakistan, research used provincial cross-sectional data (MICS), using PSM, and found that children with health insurance experience significantly better health outcomes compared with those without [16]. A study in China using the instrumental variable approach and PSM, taking cross-sectional data from the China Health and Nutritional Survey (CHNS), also indicated a substantial positive effect of health insurance on children’s health, with the impact being notably stronger among urban children compared with their rural counterparts [68]. Another study from rural China, utilizing cross-sectional data (named the China Family Panel Survey (CFPS)), using the PSM approach, depicted that a new rural cooperative medical insurance scheme has a positive impact on the health status of the poor population [69]. Similarly, another study from China assessed the causal relationship between health insurance and child health, and also used cross-sectional data CFPS-2018, using machine learning PSM, and found that rural and urban residents’ basic medical insurance has a positive impact on children’s health [70]. Additionally, research in West Africa using World Bank time series data applying the generalized least squares (GLS) method, showed that voluntary medical insurance programs significantly increase child life expectancy at birth and reduces both infant and under-five mortality rates [71]. In Nigeria, a study took the Nigerian Demographic and Health Survey 2003 cross-sectional data, employed the difference-in-difference (DID) approach, and depicted that a national health insurance program is found to improve birth weight and increase the likelihood of children receiving vaccinations for polio and diphtheria. The study highlighted that the National Health Insurance Scheme (NHIS) plays a crucial role in enhancing children’s health outcomes [72]. Moreover, research across 32 Sub-Saharan African countries taking demographic and health survey datasets of respective countries, using several quasi-experimental techniques such as DID, regression discontinuity design, instrumental variable approach, and PSM, revealed that maternal enrollment in health insurance greatly reduces the incidence of low weight and stunting in children [73].

### Strengths and Limitations of the Current Investigation

This study is among the few in Pakistan to explore the causal relationship between health insurance coverage and child nutrition status. Utilizing data from the PDHS, a large nationally representative dataset, this research stands out as a comprehensive analysis of the health effects of insurance on child nutrition in contrast with smaller studies conducted at the district or division level. The findings and scope of this investigation can be generalized, offering valuable insights for evaluating the genuine impact of health insurance and its implications for policy recommendations. However, this study has certain limitations. The cross-sectional nature of the data restricts the researchers from establishing a definitive causal link between independent and dependent variables. Although the research effectively assesses the nutritional status of insured children in Pakistan, the improvements observed may not apply to other countries. Furthermore, the analysis is constrained by variables available within the dataset. The cross-sectional format of the PDHS datasets limits the ability to track changes over time for individual children. Additionally, the survey data do not capture the complete relationships between child nutrition status and health insurance. Including other variables that are not included in this study, such as purchasing power, family income, parental education, and father insurance status, and various environmental factors like flooding, famine, and food insecurity, could provide more robust insights into the determinants of child malnutrition. But nutritional supplementation is an essential element for child growth standards. So, policies related to other variables, such as socio-economic or health insurance, will work effectively when continuous nutritional support is provided by the government. The use of a wealth index to represent purchasing power and household income may also restrict the findings, as important variables could be overlooked in both models.

The percentage of insured children was less in PDHS 2017–18; a follow-up study for the coming PDHS data will be valuable to validate the finding more accurately. The most important limitation of this study is that we applied cross-sectional PSM, which may limit generalizability and unmeasured confounders, and reserve causality issues. Moreover, the cross-sectional PSM cannot entirely eliminate unobserved confounding or guarantee true causality. Moreover, certain variables in both logistic models such as mother employment, child diarrhea, and maternal BMI could be endogenous and can be potentially influenced by the same unobserved conditions that lead a family to purchase health insurance, thus not fully isolating the independent effect of health insurance.

## 5. Conclusions

The PSM evidence supports a causal relationship between health insurance and child nutritional outcome, given the cross-sectional nature of this study and potential omitted variables. Insured children exhibit significantly better nutritional status compared with their non-insured counterparts, with a significance level of 1%. However, the cross-section cannot entirely capture the true causality. The logistic regression results confirm that health insurance coverage, higher wealth status, maternal education, access to improved water and sanitation facilities, normal maternal BMI, and urban residency all contribute to reducing the likelihood of malnutrition in children. Moreover, the logistic regression results for covariates of health insurance depicted that factors such as higher birth order, low maternal BMI, lack of access to improved water and sanitation, lower wealth status, women’s employment and education levels, and child illnesses like diarrhea and malnutrition are associated with a higher likelihood of obtaining health insurance coverage. To address these issues, the government should focus on improving marginalized communities’ socio-economic conditions and providing continuous nutritional supplementation support, which are essential for reducing malnutrition. Ensuring health insurance coverage in underprivileged areas is crucial, and efforts should be made to raise awareness about the benefits and the use of health insurance, particularly the Sehat Sahulat national health insurance program. Moreover, as conditions on insurance use by governments may limit the family to have maximum benefit from coverage, limitations should be lifted, so that registered families can use their health insurance for every health-related issue. As Pakistan has acknowledged the necessity of health insurance, the program remains inconsistent, faces political challenges (especially changes of central governments, either beneficiaries or services change), and offers limited access to other critical health services. Therefore, it is recommended that child nutritional coverage should be included as a distinct component of Pakistan’s national health insurance program.

## Figures and Tables

**Table 1:** Descriptive explaining the links between various socio-economic demographics concerning CIAF (child malnutrition).

Variable	Frequency	Percentage	*p*-Value
Sex of Child			
Female	887	21.66%	0.67
Male	921	22.49%	
Child Age in Months			
0–12 Months	284	6.94%	*p* < 0.001
13–24 Months	325	7.94%	
25–36 Months	423	10.33%	
37–48 Months	427	10.43%	
48–60 Months	349	8.52%	
Child Birth Order Number			
≤2 Years	766	18.71%	*p* < 0.001
3–4 Years	548	13.38%	
5–7 Years	378	9.23%	
Greater than 7 Years	116	2.83%	
Child Had Diarrhea Recently			
No	1435	35.04%	*p* < 0.01
Yes	373	9.11%	
Place of Residence			
Rural	1074	26.23%	*p* < 0.001
Urban	734	17.92%	
Region			
Punjab	297	7.25%	*p* < 0.001
Sindh	416	10.16%	
KPK	288	7.03%	
Balochistan	305	7.45%	
FATA	189	4.62%	
Gilgit Baltistan	108	2.64%	
Islamabad Capital	63	1.54%	
Azad Jammu and Kashmir	142	3.47%	
Source of Drinking Water			
Unimproved	1430	34.92%	*p* < 0.001
Improved	378	9.23%	
Form of Sanitation Facility			
Unimproved	1284	31.36%	*p* < 0.001
Improved	524	12.80%	
Mother’s Body Mass Index			
Less than 18.5 kg/m^2^ or Underweight	181	39.62%	*p* < 0.001
≥18.5 kg/m^2^ or Normal	1615	4.44%	
Mother’s Education			
Illiterate	1146	27.99%	*p* < 0.001
Primary	234	5.71%	
Secondary	290	7.08%	
Higher	138	3.37%	
Womens’ Working Status			
Not working	1579	38.58%	*p* < 0.001
Working	229	5.59%	
Household Wealth Index/Status			
Poorest	550	13.43%	*p* < 0.001
Poorer	507	12.38%	
Middle	324	7.91%	
Richer	254	6.20%	
Richest	173	4.22%	
Household Health Insurance Coverage			
Not Insured	1588	43.64%	*p* < 0.001
Insured	220	5.0%	

Authors’ estimation references: frequency; percentages; *p*-values.

**Table 2:** Estimations for the binary logistic model for CIAF (undernutrition among children).

Variables	Odds Ratios (*p*-Value)	95% Confidence Intervals
Biological Sex of Child		
Female (as a Reference Category)	1	-
Male	1.046 (0.49)	0.92–1.19
Child Age in Months		
0–12 Months (Reference Category)	1	-
13–24 Months	1.58 (0.00) ***	1.29–1.94
25–36 Months	2.37 (0.00) ***	1.94–2.89
37–48 Months	2.32 (0.00) ***	1.89–2.83
48–60 Months	1.93 (0.00) ***	1.56–2.36
Child Birth Order Number		
≤2 Years (Reference Category)	1	-
3–4 Years	1.21 (0.01) ***	1.04–1.40
5–7 Years	1.32 (0.002) ***	1.10–1.57
Greater than 7 Years	1.36 (0.04) **	1.01–1.82
Residence Type		
Rural (Reference Category)	1	-
Urban	0.79 (0.001) ***	0.69–0.91
Region		
Punjab (Reference Category)	1	-
Sindh	2.05 (0.000) ***	1.65–2.54
KPK	1.29 (0.03) **	1.03–1.61
Balochistan	2.52 (0.000) ***	1.95–3.26
FATA	1.53 (0.003) ***	1.16–2.02
Gilgit Baltistan	1.02 (0.88)	0.76–1.38
Islamabad Capital	0.97 (0.87)	0.69–1.37
Azad Jammu and Kashmir	0.90 (0.45)	0.69–1.18
Mother’s Body Mass Index		
Less than 18.5 kg/m^2^ or Underweight (Reference Category)	1	-
≥18.5 kg/m^2^ or Normal	0.78 (0.04) **	0.62–0.98
Child Had Diarrhea Recently		
No (Reference Category)	1	-
Yes	1.26 (0.008) ***	1.06–1.49
Drinking Water Source		
Unimproved (Reference Category)	1	-
Improved	0.79 (0.01) ***	0.66–0.95
Sanitation Facility Type		
Unimproved (Reference Category)	1	-
Improved	0.59 (0.000) ***	0.52–0.70
Mother’s Education		
Illiterate (Reference Category)	1	-
Primary	0.84 (0.11)	0.68–1.04
Secondary	0.75 (0.006) ***	0.61–0.91
Higher	0.51 (0.000) ***	0.39–0.66
Womens’ Working Status		
Not Working (Reference Category)	1	-
Working	1.28 (0.01) ***	1.045–1.58
Household Wealth Index		
Poorest (Reference Category)	1	-
Poorer	0.92 (0.48)	0.52–1.65
Middle	0.53 (0.000) ***	0.42–0.67
Richer	0.41 (0.000) ***	0.32–0.53
Richest	0.31 (0.000) ***	0.23–0.41
Health Insurance Coverage		
Not Insured (Reference Category)	1	-
Insured	0.18 (0.002) ***	0.06–0.55
Overall Model Significance		
Number of Total Observations = 4074	Prob > *Chi*2 = 0.0000	
LR *Chi*2 (29) = 576.25	Pseudo *R*2 = 0.1031	

Authors’ estimation references: odds proportions (in bracket: *p*-values); and confidence intervals. While significance level taken at: *** if *p* < 1%; ** if *p* < 5%.

**Table 3:** Estimations for binary logistic model test for child health insurance coverage.

Indicators	Odds Ratios (*p*-Value)	95% Confidence Intervals
Gender of Child	1.283 (0.34)	0.77–2.13
Age of Child	1.127 (0.21)	0.94–1.36
Child Birth Order Number		
≤2 Years (Reference Category)	1	-
3–4 Years	0.58 (0.15)	0.85–0.96
5–7 Years	3.58 (0.001) ***	1.74–7.38
Greater than 7 Years	5.79 (0.001) ***	1.99–16.8
Place of Residence		
Urban (Reference Category)	1	-
Rural	0.94 (0.001) ***	0.53–0.69
Region		
Punjab (Reference Category)	1	-
Sindh	0.27 (0.098) **	0.55–1.28
Balochistan	0.28 (0.02) **	0.03–2.22
KPK	4.37 (0.000) ***	1.93–9.92
Islamabad Capital	2.58 (0.07) **	1.91–7.31
Azad Jammu and Kashmir	2.05 (0.15)	0.76–5.55
Gilgit Baltistan	8.64 (0.000) ***	3.58–20.8
Mother’s Body Mass Index		
≥18.5 kg/m^2^ or Normal (Reference Category)	1	-
Less than 18.5 kg/m^2^ or Underweight	1.02 (0.03) **	1.31–3.42
Child Had Diarrhea Recently		
No (Reference Category)	1	-
Yes	1.87 (0.007) ***	1.45–1.73
Child Nutritional Status (CIAF)		
Not Malnourished (Reference Category)	1	-
Malnourished	1.98 (0.001) ***	1.56–1.72
Source of Drinking Water		
Improved (Reference Category)	1	-
Unimproved	2.07 (0.02) **	1.84–5.13
Type of Sanitation Facility		
Improved (Reference Category)	1	-
Unimproved	1.96 (0.01) ***	1.41–2.26
Mother’s Education		
Illiterate (Reference Category)	1	-
Primary	0.53 (0.37)	0.61–3.90
Secondary	1.01 (0.98)	0.39–2.58
Higher	5.91 (0.000) ***	2.52–13.9
Womens’ Working Status		
Not Working (Reference Category)	1	-
Working	2.01 (0.03) **	1.07–3.81
Household Wealth Index		
Poorest (Reference Category)	1	-
Poorer	0.58 (0.27)	0.22–1.53
Middle	0.94 (0.91)	0.34–2.64
Richer	1.06 (0.09) **	1.34–3.26
Richest	1.03 (0.06) **	1.31–3.46
Overall Model Significance		
Number of Observed Data = 3741	Prob > *Chi*2 = 0.0000	
LR *Chi*2 (25) = 122.95	Pseudo *R*2 = 0.1809	

Authors’ estimation references: odds proportions (in bracket: *p*-values); and confidence intervals. While significance level taken at: *** if *p* < 1%; ** if *p* < 5%.

**Table 4:** Sample matching results.

Samples	Unmatched Sample	Matched Sample	Total
Untreated/Control	198	2650	2848
Treated	3	890	893
Total	201	3540	3741

Source: Authors’ estimations.

**Table 5:** Examination for matching method quality.

The Samples/Trials	The Unmatched/Unequal	From Nearest Neighborhood Matching
Ps *R*2	0.190	0.015
LR *Chi*2	129.15	21.34
*p* > *Chi*2	0.000	0.724
Mean bias	21.4	10.7
Med bias	16.5	10.2

Source: Authors’ estimations.

**Table 6:** Covariate balance among treated and control groups (before and after matching).

Variables	Unmatched and Matched	Mean (Treated)	Mean (Control)	Bias Percentage	Reduction Percentage	*t*-Test Value	*p*-Value
Socio-Economic Variables							
Household Wealth							
Poorer	Unmatched	0.161	0.239	−19.3	61.9	−1.48	0.138
	Matched	0.161	0.191	−7.4		−0.45	0.656
Middle	Unmatched	0.206	0.196	2.3	−215.8	0.19	0.848
	Matched	0.206	0.196	7.3		0.43	0.666
Richer	Unmatched	0.205	0.188	4.2	−160.5	0.35	0.724
	Matched	0.205	0.162	11.0		0.66	0.510
Richest	Unmatched	0.294	0.183	26.2	100.0	2.34	0.019
	Matched	0.294	0.294	0.0		0.00	1.000
Place of Residence	Unmatched	0.515	0.477	7.6	100.0	0.62	0.534
	Matched	0.515	0.515	0.0		0.00	1.000
Region	Unmatched	4.029	3.219	42.3	89.1	3.35	0.001
	Matched	4.029	4.117	−4.6		−0.28	0.778
Drinking Water Source	Unmatched	0.912	0.826	25.5	−2.9	1.85	0.064
	Matched	0.912	0.824	26.3		1.52	0.131
Type of Sanitation	Unmatched	0.882	0.782	27.1	85.4	2.00	0.046
	Matched	0.882	0.897	−4.0		−0.27	0.786
Maternal and Child Variables							
Gender	Unmatched	0.573	0.509	13.0	77.3	1.06	0.291
	Matched	0.573	0.589	−2.9		−0.17	0.863
Age	Unmatched	3.147	2.929	15.5	−55.7	1.26	0.209
	Matched	3.147	3.485	−24.2		−1.43	0.156
Birth Order	Unmatched	2.059	1.818	25.3	26.6	2.16	0.031
	Matched	2.059	1.882	18.6		1.05	0.296
Mother’s BMI	Unmatched	0.956	0.909	18.5	−58.9	1.32	0.186
	Matched	0.956	0.882	29.4		1.58	0.118
Mother’s Education							
Primary	Unmatched	0.103	0.143	−12.3	27.4	−0.95	0.344
	Matched	0.103	0.132	−8.9		−0.53	0.598
G1 Secondary	Unmatched	0.118	0.224	−28.4	58.4	−2.09	0.037
	Matched	0.118	0.074	11.8		0.87	0.385
Higher	Unmatched	0.455	0.152	69.8	100.0	6.87	0.000
	Matched	0.455	0.456	0.0		−0.00	1.000
Mother’s Employment	Unmatched	0.235	0.119	30.5	11.1	2.90	0.004
	Matched	0.235	0.338	−27.1		−1.33	0.187
Disease Related Variable							
Child Had Diarrhea	Unmatched	0.162	0.188	−7.1	100.0	−0.56	0.574
	Matched	0.162	0.162	0.0		−0.00	1.000

Source: Authors’ estimations.

**Table 7:** Influence of health insurance on child nutritional outcome (CIAF).

The Sample/Trials	Unmatched/Unequal	ATT
Treatment	0.3235	0.3236
Controled	0.4324	0.2868
Differences	−0.1088	0.0367 ***
Standard (SD) error	0.0606	0.0823
T-test value	−1.80	0.45

Source: Authors’ estimations. Significance level taken at: *** if *p* < 1%.

## Data Availability

This study utilized the secondary data of the Pakistan Demographic and Health Survey 2017–18. Available online at: URL: https://dhsprogram.com/data/dataset/Pakistan_Standard-DHS_2017.cfm?flag=1 (accessed on 20 October 2020).
